# MoO_3_, TiO_2_, and MoTiO_5_ based oxide semiconductor for photovoltaic applications

**DOI:** 10.55730/1300-0527.3470

**Published:** 2022-08-02

**Authors:** Fatma BAYRAKÇEKEN NİŞANCI

**Affiliations:** Department of Chemistry, Faculty of Science, Atatürk University, Erzurum, Turkey

**Keywords:** Photoelectrode, MoO_3_, TiO_2_, MoTiO_5_, SILAR

## Abstract

Topographic essential synthesis of nanomaterials by adjusting easy preparatory factors is an effective way to improve a variety of nanostructured materials. The SILAR technique is used to evaluate the manufacturing samples of MoO_3_, TiO_2_, and MoTiO_5_ nanostructures. These nanostructures of MoO_3_, TiO_2_, and MoTiO_5_ are used as electrode materials in photovoltaic systems. The link between photoelectrochemical characteristics and MoO_3_, TiO_2_, and MoTiO_5_ nanostructures is studied in depth. The photoelectrochemical characteristics of MoO_3_, TiO_2_, and MoTiO_5_ nanostructures are discovered to be highly dependent. At a 5mV/s scan rate, the photocurrent of MoO_3_, TiO_2_, and MoTiO_5_ electrodes surged fast when sunlight was turned on, reaching values of 1.03 mA cm^−2^, 1.68 mA cm^−2^, and 14.20 mA cm^−2^, respectively. As soon as the solar illumination was turned off, the photocurrent value dropped to zero. Photocurrent transitions showed a quick, homogeneous photocurrent counterpart; this suggested that charge transfer in these ingredients is speedy and possibly related to the crystal buildings of MoO_3_, TiO_2,_ and MoTiO_5_. MoTiO_5_ nano-belt and nano-disc thin films have typical uses in the photoelectrochemical sector because they have the best photoresponse and stability.

## 1. Introduction

A choice to traditional inorganic solar cells is the photovoltaic apparatus which rests upon molecular and nanostructured semiconductors. The materials which are used in these classes of apparatus have diverse head starts, such as simple making, mechanical elasticity, and the potential large-area manufacture affordable. At the same time, some other optoelectronic apparatus such as light-emitting diodes and sensors benefit from potential applications [1–3] Titanium oxide is one of the metal oxide semiconductors which have been most fairly studied and owing to its special optical, magnetic, and electrical properties, it is used in various practical applications.

Titanium dioxide (TiO_2_) may be utilized in numerous applications and it is attractive for high technology due to its amazing electrical, optical, and chemical properties; comprising vast band gap energy, antitoxic and diaphaneity in the visible light, well index of refraction, electrochemical stability, and fine isolating features [4]. Apart from these, particularly photocatalysis, photovoltaic and gas sensing have utilized titanium dioxide [5,8], and owing to its high conversion efficiency, also dye-sensitized solar cells (DSSC) have used it in the alternative energy field in which porous TiO_2_ electrodes are used [9]. On the other hand, the opportunities to use this material in the above-mentioned applications mainly regard their production cost and performance. Thus, it is an important factor to search for low-cost production technologies in these applications development in the future [10]. Beneath the distinct impression of solar radiation and photogenerated carriers, for enhancing photo-absorption of the TiO_2_-based materials, various strategies have been studied and doping with metal and nonmetal ions [11], altered with excellent metals [12], connected with other semiconductors (Bi_2_WO_6_, ZnO, MoO_3_, and CdS etc.) [13] and sensitization via various organic materials delicate to sunlight [14–16] instance can be given. For the generation of a hetero-junction structure, it has been accepted as an efficient method to couple TiO_2_ with other semiconductors to gain a better photo-catalytic movement of TiO_2_ [17].

Molybdenum oxide (MoO_3_) is among transition metal oxides and it is a large band gap n-type semiconductor material. MoO_3_, which is among the most significant stratified materials has conspicuousness in many areas such as photochromic and electrochromic devices, catalysis, energy stowage, and gas sensors due to its unique features. MoO_3_ has been implemented as a gas sensor [18]. On the other hand, studies have reported that MoTiO_5_ can act as a charge capture layer in memory devices [19]. The transfer of photo-excited electrons from the valence band (VB) of TiO_2_ to the conduction band (CB) of MoO_3_ via direct Ti/O/Mo junction and prolonging the lifetime of the photo-excited electron-hole pairs likely enhance photosensitivity [20].

Until this point in time, different strategies have been created to magnify nanoparticles of IV–VI semiconducting materials. To prepare IV–VI semiconductors, atomic layer epitaxy (ALE) [21], chemical bath deposition (CBD) [22], chemical vapor deposition (CVD) [23], and consecutive ionic layer adsorption and reaction (SILAR) [24] have been traditionally used. SILAR depends on the dip of the substrate into independently positioned cations and anions followed by washing after every response, it makes possible to give a heterogeneous response among the solid stage and the solvated ions in the solution. In order to form thin films which are uniform, compact and crystalline, it is ideal to use the SILAR method. Moreover, SILAR does not need a target or vacuum, the deposition rate and the film thickness can be easily controlled over a long range by varying the number of deposition cycles.

**Figure 1:**
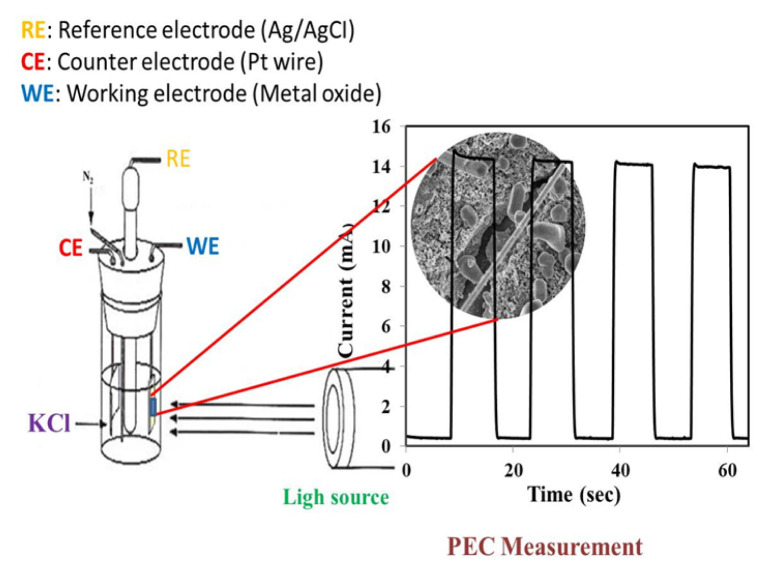
Diagrammatic of the photocurrent response of MoTiO_5_ nanostructures on an ITO.

In this study, a different new method for synthesis of MoO_3_, TiO_2_, and MoTiO_5_ nano-structures based on SILAR to indium tin oxide (ITO) electrodes is presented. MoO_3_/TiO_2_/MoTiO_5_ nano-structures obtained were recognized by scanning electron microscopy (SEM), X-ray photoelectron spectroscopy, energy dispersive spectroscopy (EDS), X-ray diffraction, and photo-current analysis. Improvement in photocurrent density between MoO_3_, TiO_2,_ and MoTiO_5_ electrodes and electrochemical impedance spectroscopy (EIS) to determine the charge transfer resistance at the interface studies have been done. It is indicated by the test results that, by setting the spool time, control of the morphology and size of MoO_3_, TiO_2_, MoTiO_5_ nano-structures can be possible. Good photovoltaic properties are shown by the nano-structured MoO_3_, TiO_2_, MoTiO_5_ photo-electrodes formed and they can be utilized in applications of solar energy conversion. Using optical-quality enlarged nano-structures with a low defect concentration which has a stable and repeatable photo-current through many cycles is the general test strategy of this study as can be given in Figure 1.

## 2. Materials and methods

MoO_3_, TiO_2_, and MoTiO_5_ nanofilms were formed by reaction SILAR method and sequential ionic layer adsorption. All of the electrolyte solutions used in this study were prepared using deionized water (i.e.>18MΩ) from a Milli-Ωsystem. In this method, 25 mL of 0.05 M Titanium III chloride (TiCl_3_) and 25 mL of 0.05 M of sodium molybdate (MoNa_2_O_4_) as cationic and 25 mL of 0.02 M of sodium hydroxide (NaOH) as an ionic pioneer were expended. Four beaker SILAR contrivance was used to prepare MoO_3_ and TiO_2_ thin films with different nanostructures, where cationic and ionic precursors were formed from two rinse steps. The cleaned ITO substrate was submersed for 20 s in a cationic solution (MoNa_2_O_4_) for the adsorption of molybdenum ions onto the substrate and in the anionic solution (NaOH) for 20 s to form MoO_3_ materials. One might as well say, the well-cleaned ITO substrate was submersed in a cationic solution (TiCl_3_) for 20 s for the adsorption of the titanium ions on the substrate and anionic solution (NaOH) for 20 s to form TiO_2_ materials. A six-beaker SILAR system was expended to arrange MoTiO_5_ nanostructured thin films, where cationic and ionic solutions were individuated by a rinse step. The cleaned ITO substrate was submersed in a cationic solution (MoNa_2_O_4_) for 20 s for the adsorption of molybdenum ions on the substrate and a cationic solution (TiCl_3_) for 20 s for the adsorption of the titanium ions on the substrate. The ITO substrate was washed in deionized water for 10 s to remove the loosely bound molybdenum, titanium, and hydroxide ions. Washing the substrate using deionized water for 10 s again distinguish surplus or nonreaction ions. In this manner, a SILAR cycle of accumulating each of MoO_3_, TiO_2_, MoTiO_5_ is finished. This type of 80 SILAR period was reiterated to obtain the optimum thickness of MoO_3_, TiO_2_, and MoTiO_5_ thin films.

### 2.1. Analytical methods

An electrochemical workstation (attached to a three-electrode cell, BAS 100B/W) was exercised for photoelectrochemical tests (Chrono-Amperometry measurements). Cyclic voltammetry (CV) and electrochemical impedance spectroscopy (EIS) experiments were performed with Gamry (600+) potentiostat systems connected to a three-electrode cell. ITO-coated quartz (10 Ω cm^−2^) was used as the working electrode for the electrochemical measurements and the photoelectrochemical measurements. The counter and reference electrodes that were used included a Pt wire and Ag/AgCl (saturated KCl), respectively. The photocatalytic performance of MoO_3_, TiO_2,_ and MoTiO_5_ electrodes was determined by performing cyclic voltammetry studies for the one-electron reduction of reversible Fe(CN)_6_^3−^/Fe(CN)_6_^4−^ redox system in a solution containing 10.0 mM K_3_Fe(CN)_6_ and 0.1 M KCl. X-ray diffraction plots of the deposited films were registered with a Rigaku powder X-ray diffraction meter with a CuK X-ray source (λ = 1.5406 Ả). Morphological workout and identification of the elemental composition (Mo/O), (Ti/O), and (Mo/Ti/O) of MoO_3_, TiO_2_, MoTiO_5_ nanostructures were performed by an EDS united with a scanning electron microscope (ZEISS system). X-ray photoelectron spectroscopy (XPS, Spect-Flex spectrometer) measurements of metal oxide samples were obtained by using a standard Al X-ray source. Atomic force microscopy images of electrodeposits were acquired in ambient conditions, with a Hitachi 5100N instrument. Ultraviolet-visible (UV-Vis) spectroscopy measurements were obtained from a Shimadzu UV-3600 Plus spectrophotometer. Photoelectrochemical quantifications of MoO_3_, TiO_2_, and MoTiO_5_ nanostructures were made at room temperature using 0.1 M KCl solution. The photocurrent intensity was enrolled under illumination by AM 1.5 G (1 sun, 100 mW/cm^2^) exploiting a solar simulator (SolarLight-16 S).

## 3. Results

**Figure 2:**
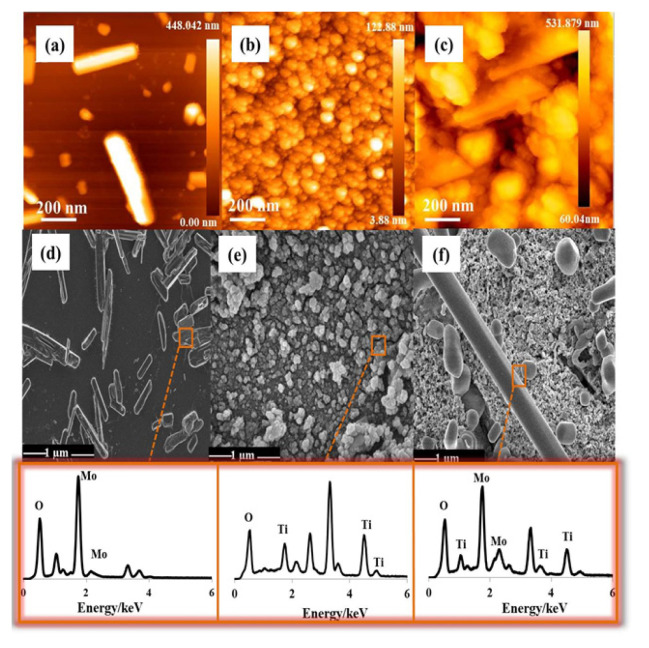
AFM images of (a) MoO_3_, (b) TiO_2_ and (c) MoTiO_5_ nanostructures on ITO electrode. SEM and EDS micrographs of (d) MoO_3_ on ITO electrode, (e) TiO_2_ on ITO electrode, and (f) MoTiO_5_ nanostructures on ITO electrode.

Figure 2 indicates the AFM and SEM images of the coated electrode surface and it emphasizes the tremendously special surface of MoO_3_, TiO_2_, and MoTiO_5_ film. Controlling metal oxides synthesized by the SILAR method on the substrate (Figure 2), its ability to easily adjust the shape and size of MoO_3_, TiO_2_, and MoTiO_5_ nanostructures has been demonstrated by the results and SEM and AFM images. Pure MoO_3_ nano-belts at low and high magnification are shown in Figures 2(a)–2(d) and MoO_3_ is seen as scattered nano-belts about 500 nm long and 50 nm thick. Figures 2(b)-2(e) show AFM and SEM micrographs of TiO_2_ nanostructures with diameters of approximately 50 nm shaped as nano-discs. While its morphology owns great changes when comparing the TiO_2_ and MoO_3_ precursor, TiO_2_ nanoparticles in nano-disc shape own nano-belts structures on the MoO_3_ surface. SEM and AFM data (Figures 2c–2f) show MoTiO_5_, the combined structures of nano-belts and nano-discs. With the increase of photo-absorption efficiency on MoTiO_5_ nanostructured thin films, which have impressive photosensitivity, light absorption, reflection and scattering can be increased significantly. The SEM image of MoTiO_5_ can be seen in Figure 2(f) and the results indicate that the MoTiO_5_ nanostructures [25] were designed perfectly after the SILAR procedure. Well connection between MoO_3_ nanoparticles in nano-belts shape and TiO_2_ nanoparticles in nano-disc shape can be observed. Additionally, to verify the elements’ composition, the EDX analysis of the MoTiO_5_ was performed and the results are given in Figure 2(f). The existence of Ti, Mo, and O atoms in the region elected and each component’s respective heaviness proportions being 52.98% (O), 17.14% (Mo), and 19.63% (Ti) can be seen.

**Figure 3:**
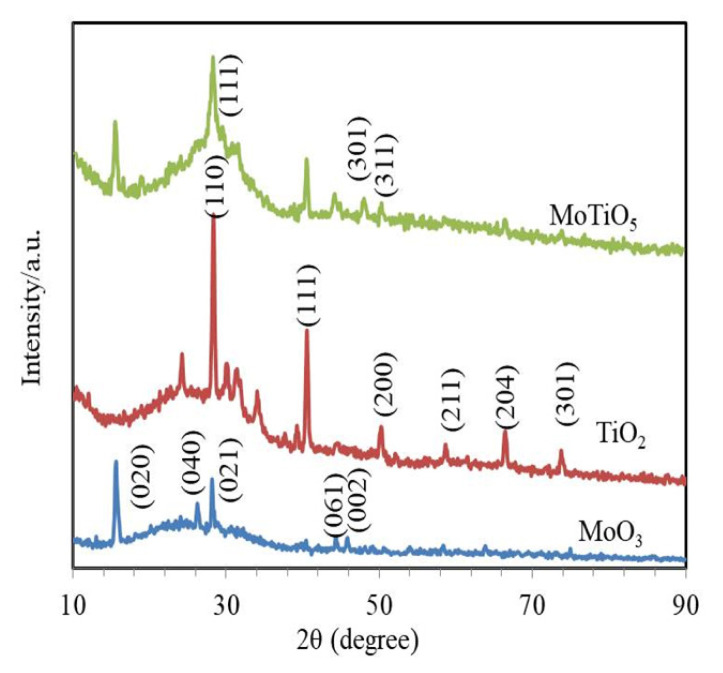
The X-ray diffraction (XRD) exemplary of TiO_2_, MoO_3,_ and MoTiO_5_.

To examine the crystal growth of the as-designed samples, a systematic XRD study was performed and Figure 3 shows the results. The diffraction peaks with reference to the (101), (111), (2 0 0), (2 1 1), (2 0 4), and (301) crystal orientations with the lattice constants a = 3.755 Å and c = 9.5114 Å confirm the tetragonal anatase phases of the TiO_2_ nanoparticles in compliance with the JCPDS file 21-1272 [26]. Regarding the unalloyed MoO_3_, the XRD planes displayed at 2θ = 12.8°, 25.8°, 38.9°, 46.1°, and 49.3° correspond to the (020), (040), (021), (061), and (002) orientations of orthorhombic construction MoO_3_ with the JCPDS Card no. 05-0508 and the lattice literals a 5 3.96 Å and c 5 3.7 Å [27]. It was shown in the sample that nano-belts average thickness is approximately 50 nm. The MoTiO_5_ sample exhibited diffraction peaks at 28.3**°**, 47.1°, and 49.9°, which MoTiO_5_ had phases with peaks at (101), (301), and (311), respectively. From the MoTiO_5_ curve [28], it can be well observed that the diffraction peaks of MoTiO_5_ can be successfully prepared by this process.

**Figure 4:**
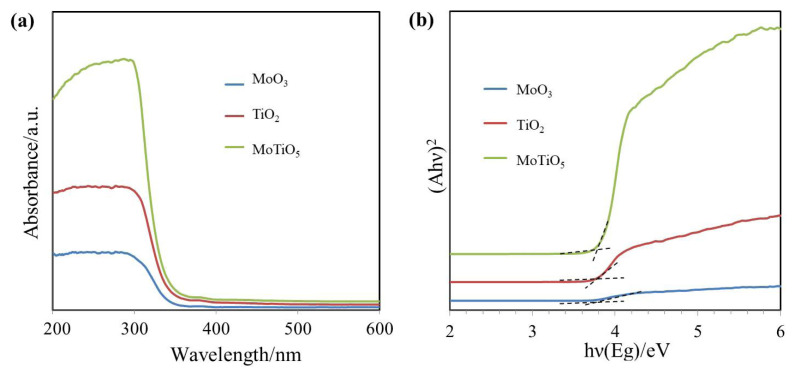
UV-vis absorption spectrum (a) and plots of *(Ahν)^2^* versus *(hν)*, (b) of MoO_3_, TiO_2_, MoTiO_5_.

Figure 4(a) indicates UV–visible absorption spectra of arrant TiO_2_, MoO_3,_ and MoTiO_5_. In Figure 4a, it is clearly seen that the absorption intensity in MoTiO_5_ is higher at about 300 nm compared to TiO_2_ and MoO_3_. The band gap values of the films were calculated by plotting *(Ahν)^2^* vs. *(hν)* and extrapolating the linear portion of the graph to the energy axis (Figure 4b) [29]. The plots of *(Ahν)^2^* versus *(hν)* are illustrated in Figure 4(b), the band gaps of the synthesized MoO_3_, TiO_2,_ and MoTiO_5_ thin films were found to be 3.0 eV, 3.2 eV, and 3.6 eV, respectively. As can be seen, among the developed materials, MoTiO_5_ is one of the semiconductor metal oxides with a wide band gap of 3.6 eV.

**Figure 5:**
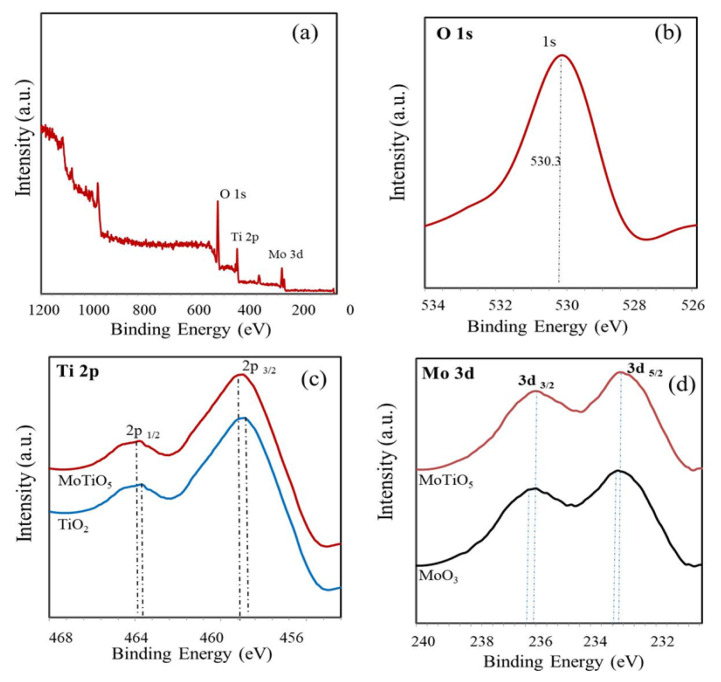
XPS interpretation (a) The large scan XPS spectrum of the MoTiO_5_, (b) O 1s of the MoTiO_5_, (c) Ti 2p of TiO_2_ and MoTiO_5_, (d) Mo 3d of MoO_3_ and MoTiO_5_.

XPS study was performed to indicate the presence of Mo/Ti/O bond in the nano-composites and Figure 5 shows the results. It is shown in Figure 5(a) that it is shown in the large-scan XPS spectrum that the overborne C. Ti, O, and Mo elements are formed through patterns among these elements and the C element is from the XPS instrument itself, and no other elements are appointed. The high-definition XPS spectrum of O 1s and the location of the binding energy of 530.3 eV are shown in Figure 5(b). O 1 s spectra indicate, that the O–H content increases with respect to the O lattice in Mo–O and Ti-O, thus surface hydroxyl groups are the primary source of protons. It is shown in Figures 5(c) and 5(d), for TiO_2_ that the binding energies of Ti 2p1/2, and Ti 2p3/2 peaks are detected at 464.3 eV and 458.5 eV, and the binding energies of Mo 3d3/2 and Mo 3d5/2 peaks are detected at 236.4 eV and 233.6 eV, sequentially. In contrast, in case of the MoTiO_5_, it is reported that the binding energies of Ti 2p1/2, and Ti 2p3/2 peaks are detected at 464.2 eV and 458.9 eV, and the binding energies of Mo 3d3/2 and Mo 3d5/2 peaks are detected at 236.1 eV and 233.4 eV, sequentially. With the creation of MoTiO_5_ increase in the binding energies of Ti 2p and a decrease in Mo 3d can be observed. As a result, the bonds formed by Ti oxidation and Mo reduction are the main reasons for the excellent photocurrent property. Thus, it is implied that strong interaction exists between Mo/O/Ti and MoTiO_5_ is generated on ITO [30].

**Figure 6:**
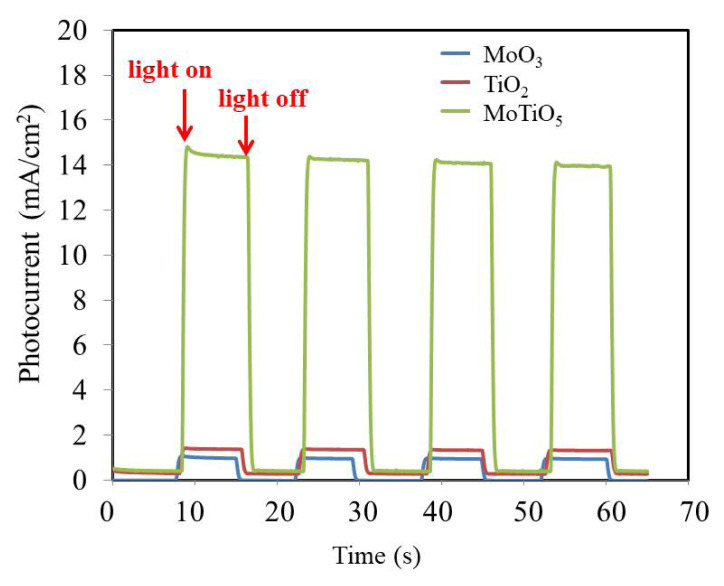
Photocurrent counterpart of the MoO_3_, TiO_2,_ and MoTiO_5_ nanofilms on ITO electrodes.

Figure 6 shows the transient photocurrent of MoO_3_, TiO_2,_ and MoTiO_5_ nanostructured electrodes produced under chopped one-sunlight illumination. Chrono-Amperometry measurements were carried out in 0.1 M KCl electrolyte under the irradiance of 100 mW/cm^2^ from SolarLight-16 S for Pt counter and Ag/AgCl reference electrodes. The photocurrent counterpart was measured in 8 s on-off cycles at the short-circuit potential in 0.10 M KCl electrolyte solution without compromising reactive or co-catalysts. When exposed to solar illumination, photocurrents of MoO_3_, TiO_2,_ and MoTiO_5_ electrodes rise to 1.03 mA cm^−2^, 1.68 mA cm^−2^, and 14.89 mA cm^−2^, respectively. When there is no solar illumination, the photocurrent value drops to zero.

Figure 6 depicts the quick and monotonic photocurrent in these transients and the rapid change transport mechanism and its connection to single crystalline MoTiO_5_. Furthermore, the photocurrents produced by the MoTiO_5_ nanostructures are constant and reproducible across several cycles, showing that the electrode is photocorrosion-free. The chrono-amperometry study present in Figure 6, also supports the results obtained from CV and EIS measurements. Moreover, it is also beneficial for their photocatalytic performance that the peerless structure of MoTiO_5_ would make the light storage better [31,32].

**Figure 7:**
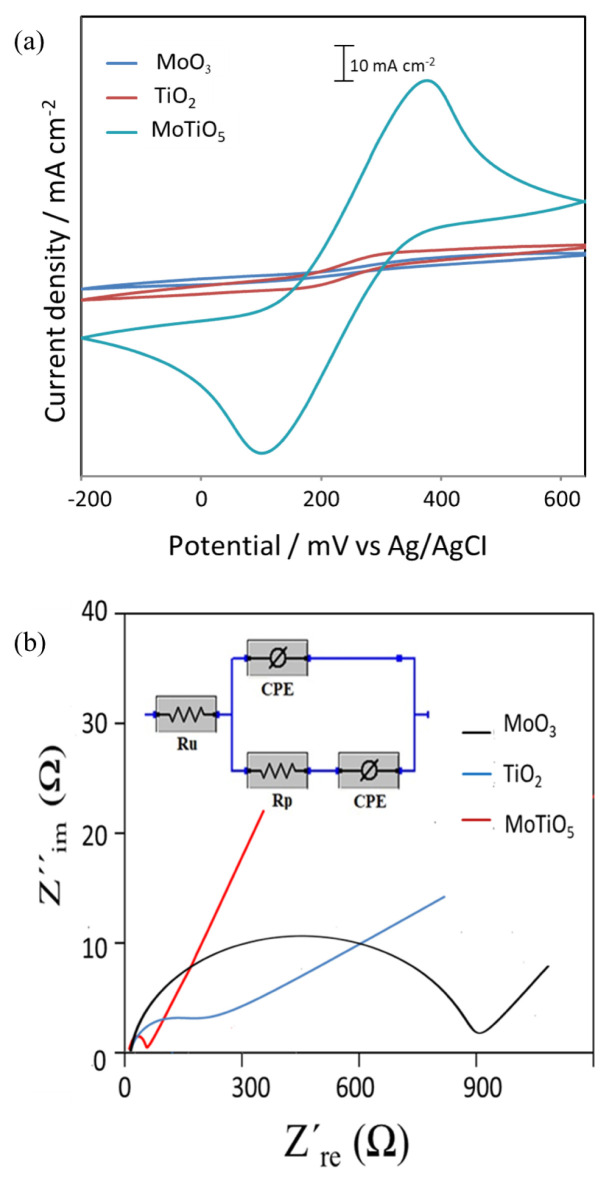
(a) CV curves, (b) Nyquist plots of metal oxide samples in a solution containing 10 mM K_3_Fe(CN)_6_, 10 mM K_4_Fe(CN)_6_, and 0.1 M KNO_3_ of MoO_3_, TiO_2_, MoTiO_5_. Inset: The equiv. circuit model. Frequency range: 0.1−105 Hz.

Electrochemical performances of MoO_3_, TiO_2_, and MoTiO_5_ materials were investigated by using CV and EIS techniques. CVs obtained in an electrochemical solution containing 0.1 M KNO_3_ and 10 mM Fe(CN)_6_^3−^/Fe(CN)_6_^4−^ for MoO_3_, TiO_2_ and MoTiO_5_ prepared in metal oxide materials are shown in Figure 7a. While the electrochemical activity of MoO_3_, and TiO_2_ materials was quite low, the electrochemical activity of MoTiO_5_ improved as seen in Figure 7a. Nyquist graphs obtained for TiO, MoO_3,_ and MoTiO_5_ composite films prepared on ITO electrode in 0.1 M KCl solution containing 10 mM Fe(CN)_6_^3−^/Fe(CN)_6_^4−^ are shown in Figure 7b. Nyquist graphs are fitted according to the electrical circuit given in Figure 7b. Here, the faradaic electron transfer resistance (Rp) corresponds to the diameter of the formed semicircle. The solution resistance (R_w_) is the intersection point of the Real Z′ axis of the graph [33]. The stationary phase element (CPE) is the capacitance of the double layer [34]. Accordingly, electron transfer resistances for MoO_3_, TiO_2,_ and MoTiO_5_ composite films were determined as 862 Ω, 179 Ω, and 10 Ω, respectively. When the electron transfer values obtained were evaluated, it was determined that the electron transfer resistance was quite low since the electrochemical activity of the MoTiO_5_ composite film was the highest. Thus, the impedance study carried out also supported the photocurrent studies.

## 4. Discussion

In conclusion, the structure of MoO_3_, TiO_2_, and MoTiO_5_ nanostructures was effectively altered using the SILAR approach by adjusting basic preparation conditions. Experiments also indicated that altering the deposition time may change the size of MoTiO_5_ nanostructures. MoTiO_5_ nanostructures, photocurrent measurements demonstrated a decreased fault concentration and higher optical quality. The photocurrent produced by the MoTiO_5_ nanostructures is steady and reproducible over many cycles, showing that the electrode is photocorrosion-free. The characterization methods and photoelectrochemical studies on these metal oxides have shown that the method applied in the synthesis of these materials affects the crystal structure and grade of the materials at the same time, the nanosize of the synthesized material has a direct effect on the electronic properties and performances of these materials.
